# Effects of Forest Therapy on Depressive Symptoms among Adults: A Systematic Review

**DOI:** 10.3390/ijerph14030321

**Published:** 2017-03-20

**Authors:** Insook Lee, Heeseung Choi, Kyung-Sook Bang, Sungjae Kim, MinKyung Song, Buhyun Lee

**Affiliations:** 1 Faculty of College of Nursing, The Research Institute of Nursing Science, Seoul National University, Seoul 03080, Korea; lisook@snu.ac.kr (I.L.); ksbang@snu.ac.kr (K.-S.B.); sungjae@snu.ac.kr (S.K.); 2 College of Nursing, Seoul National University, Seoul 03080, Korea; mk0408@snu.ac.kr (M.S.); bhyunlee@snu.ac.kr (B.L.)

**Keywords:** systematic review, forest therapy, depression, adults

## Abstract

This study systematically reviewed forest therapy programs designed to decrease the level of depression among adults and assessed the methodological rigor and scientific evidence quality of existing research studies to guide future studies. This systematic review was conducted in accordance with the Preferred Reporting Items for Systematic Reviews and Meta-Analyses guidelines. The authors independently screened full-text articles from various databases using the following criteria: (1) intervention studies assessing the effects of forest therapy on depressive symptoms in adults aged 18 years and older; (2) studies including at least one control group or condition; (3) peer-reviewed studies; and (4) been published either in English or Korean before July 2016. The Scottish Intercollegiate Guideline Network measurement tool was used to assess the risk of bias in each trial. In the final sample, 28 articles (English: 13, Korean: 15) were included in the systematic review. We concluded that forest therapy is an emerging and effective intervention for decreasing adults’ depression levels. However, the included studies lacked methodological rigor. Future studies assessing the long-term effect of forest therapy on depression using rigorous study designs are needed.

## 1. Introduction

Forest therapy or “forest bathing” refers to visiting a forest or engaging in various therapeutic activities in a forest environment to improve one’s health and wellbeing [1,2]. Societies have been urbanizing rapidly and more people reside in an urban environment with limited access to nature; therefore, diverse efforts including political and landscaping efforts have been made to make nature more accessible [3]. With an increasing awareness of health benefits of forest therapy, it has been implemented on diverse population [1]. Particularly, the psychological benefits of forest therapy have received special attention as people residing in urban environments have been reported to be at an increased risk of prolonged exposure to stressful situations and mental health problems [4,5,6]. Compared to control groups, forest therapy significantly improves adults’ mental health by decreasing stress, depression, anxiety, and anger levels [7].

A systematic review summarizes the results of the available research studies and provides synthesized evidence on the effectiveness of those studies [8]. It enables researchers to identify the current state of the science, areas for future researchers to improve upon, and provides strong evidence for up-to-date practices and policy developments [9]. It is also beneficial for emerging topics that require systematic evaluation and synthesis of the evidence quality (e.g., feasibility and effectiveness of intervention) as well as well-established areas of research with accumulated scientific evidence that need be updated regularly.

Despite the increased attention to the various health benefits of forest therapy, until now, systematic reviews of the body of evidence for the effectiveness of forest therapy on mental health have not been conducted. A clearer and comprehensive understanding of the effectiveness of forest therapy on mental health is important for further refinement of forest therapy programs. Among the several mental health outcomes included in the forest therapy research, our paper will focus on depression. Depression is the leading cause of disability; approximately 350 million (5% of the world’s population) suffer from this debilitating disorder [10]. The specific aims of this study were to: (1) provide a broad overview and synthesize the evidence on the usefulness of forest therapy to improve the level of depressive symptoms in adults; and (2) assess the methodological rigor and scientific evidence quality of existing research studies to guide future studies evaluating the effects of forest therapy on adults’ experiencing depressive symptoms. In the present review, forest therapy was defined as visiting a forest or engaging in various therapeutic activities in a forest environment to improve one’s health and wellbeing [1,2].

## 2. Methods

### 2.1. Literature Search

This systematic review was conducted in accordance with the Preferred Reporting Items for Systematic Reviews and Meta-Analyses (PRISMA) guidelines. We searched PubMed, EMBASE, Cumulative Index to Nursing and Allied Health, PsycARTICLES, Korean Studies Information Service System, Research Information Sharing Service, and DBpia to identify relevant studies published until July 2016. The search terms were chosen from the USNLM Institutes of Health list of Medical Subject Headings for 2015. Search terms included “trees”, “forests”, “wood”, “affect”, “depression”, “emotions” and “depressive disorder”. Search terms used to identify relevant studies for the review are listed in Appendix A, Table A1.

### 2.2. Selection Criteria

The initial eligibility assessment was conducted by one author by reviewing the title and abstracts. Then, two authors (MinKyung Song and Buhyun Lee) independently screened the full text versions of 66 articles using the following criteria: (1) intervention studies assessing the effects of forest therapy on depression among adults aged 18 years and older; (2) studies including at least one control group or condition; (3) peer-reviewed studies; and (4) been published either in English or Korean.

### 2.3. Data Extraction

The four authors (Heeseung Choi, Kyung-Sook Bang, MinKyung Song, and Buhyun Lee) independently performed the data extraction. The following data were extracted from each study: first author, date and place of publication, study design, sample size, setting, ethical consideration, participants’ characteristics, number of participants enrolled, summary of the intervention and control conditions, measures, reported outcomes, and risk of bias. The extracted data were input into standardized MS word (Microsoft Corporation, Seattle, WA, USA) files. Any disagreements were resolved by discussion between the authors.

### 2.4. Quality Assessment Tool

The Scottish Intercollegiate Guideline Network (SIGN) measurement tool (Healthcare Improvement Scotland, Edinburgh, Scotland) was used to assess the risk of bias in each study included in this review. The SIGN was developed in 1993 to improve the quality of health care for patients in Scotland by reducing the variation in practice and outcome, through the development and dissemination of national clinical guidelines containing recommendations for effective practice based on current evidence [11]. Using the SIGN, we evaluated the internal validity and risk of bias of the study and assigned values of “high quality (++),” “acceptable (+),” “low quality (−),” or “unacceptable—reject (0)” to each study. The risk of bias was evaluated independently by four reviewers (Heeseung Choi, Kyung-Sook Bang, MinKyung Song, and Buhyun Lee) and any disagreements were resolved through a consensus process.

## 3. Results

### 3.1. Study Identification and Selection

**Figure 1:**
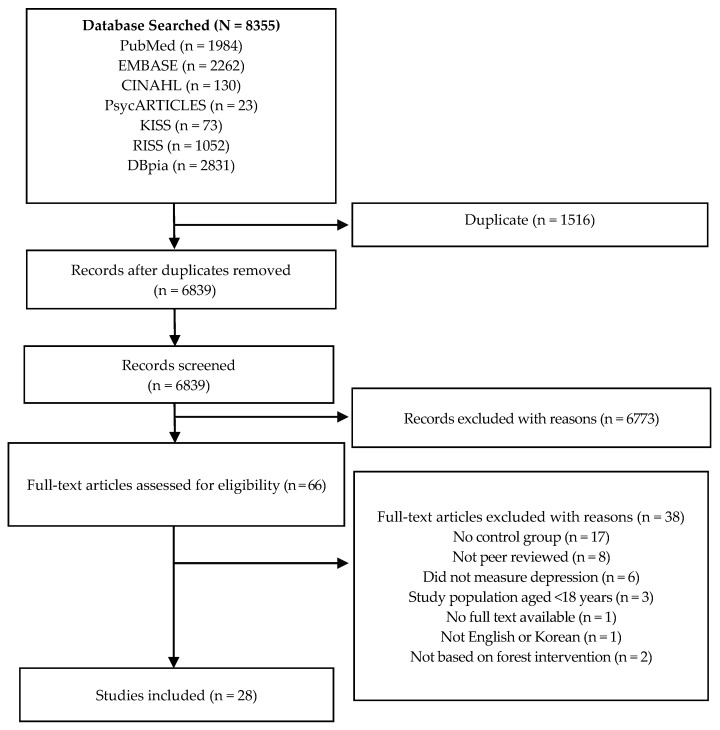
Preferred Reporting Items for Systematic Reviews and Meta-Analyses (PRISMA) Flow Diagram of the Screening Process.

Records (N = 8355), including 4399 records published in English and 3956 records published in Korean, were retrieved from the initial database searches. These search results were imported using EndNote X7 and 1516 duplicates (1356 English articles and 160 Korean articles) were removed. A detailed flow diagram of the screening process is shown in Figure 1. After excluding an additional 6773 records based on the review of the study titles and abstracts, the remaining 21 articles published in English and 45 articles published in Korean were assessed for eligibility. Many articles were excluded because those studies addressed topics that were not relevant to forest therapy, such as tree analysis (i.e., classification and regression tree analysis), biliary/bronchial tree, forest modeling, and forest fragmentation. Finally, 28 articles (English: 13, Korean: 15) were included in the present systematic review.

### 3.2. Study Characteristics

The general characteristics of the studies included in this review are summarized in Table 1. And summary of the studies included in this review are presented in Table 2 and Table 3. The selected studies were published between 1996 and 2016, and 24 of the 28 studies were published within the last five years. All the studies were conducted in Asian countries (Korea, Japan, and China) except one, which was conducted in the United Kingdom. Sixteen studies were conducted on healthy adults and the rest of the studies (n = 12) were conducted on adults with various health problems such as hypertension, cancer, and mental disorders. Among the 12 studies conducted for adults with health problems, six studies targeted psychiatric patients [12,13,14,15,16,17]; however, only one study [17] was conducted with patients with major depressive disorder. Two studies [12,15] were conducted for psychiatric patients with various diagnoses such as substance use disroders, schizophrenia, other psychotic disorders, mood disorders, and anxiety disorders. These two studies, however, did not specify what percentage of their samples were patients with major depressive disorder. Other studies targeted specific diagnoses such as Hwa-Byung [13], neurocognitive disorders [14], and alcoholism [16].

Regarding the study design, 11 studies used a crossover trial design and only six of the studies [16,18,19,20,21,22] used a randomized controlled trial (RCT) design. Four out of six RCT design studies were conducted with adults with health problems. The most common types of control condition used in the non-equivalent control group design studies were “normal daily routines” for healthy adults (five out of six studies) and “treatment-as-usual” for adults with health problems (three out of five studies).

The sample size ranged from 11 [23] to 92 [16]; for almost 43% of the studies, the sample size was less than 20. Furthermore, about one third of the studies (28.5%) did not follow the ethical protocol such as being reviewed and approved by the Institutional Review Board (IRB).

#### 3.2.1. Format and Content of Forest Therapy

The forest therapy programs tested in these 28 studies varied in terms of format and content of the programs. The length of time that the interventions were undertaken ranged from one day to twelve weeks. The duration of the forest therapy ranged from twelve minutes [29] to three hours [33]. About one third of the studies [23,26,27,28,29,32,34,39] offered forest therapy programs and control conditions (such as activities in downtown) every other day during the two-day periods. Three studies [12,21,25] used a one-time intervention that lasted a few hours to half a day. One study did not report duration details of the intervention [36].

Regarding the content of forest therapy, walking in the forest was the key component of the forest therapy that was included in most studies except one [28]. Other therapeutic activities included in forest therapy programs were experiencing forest through the five senses (seeing, hearing, touching, smelling, and tasting), forest viewing, forest meditation, Qi-Qong, aromatherapy, herbal tea therapy, and craftwork using natural materials.

#### 3.2.2. Depression Measures

The most commonly used self-report measure for depression in these 28 studies was the Profile of Mood States (POMS). For articles published in English, nine studies [12,19,21,22,25,28,29,34,39] used POMS to assess the level of depression and three studies [28,29,39] used Semantic differential (SD) method. Other scales used by the studies included the Hamilton Rating Scales for Depression [20], Beck Depression Inventory (BDI) [16,20,24,31], positive and negative affect schedule (PANAS) [34]. For studies published in Korean, the POMS [15,23,26,27,32,38] and BDI [13,14,15,17,18,35] were the most commonly used scale. Other scales used to measure depression were the Hospital Anxiety and Depression Scale (HADS) [38], Hamilton Rating Scales for Depression [17], Montgomery-Asberg Depression Rating Scales [17], Symptom Check List (SCL-90-R) [32], and Zung Self-Rating Depression Scale (SDS) [36]. Fourteen studies [13,15,17,18,19,21,22,23,24,25,26,28,29,39] used both self-report, paper-and-pencil questionnaires, and physiological measures, while 14 studies [12,14,16,20,27,30,31,32,33,34,35,36,37,38] used only self-report, paper-and-pencil questionnaires. Physiological or objective measures included heart rate variability (HRV), blood pressure, heart rate, and amylase concentration. Detailed information of the measures included in these studies is summarized in Table 2 and Table 3.

#### 3.2.3. Effects of Forest Therapy on Depression

All 28 studies assessed the level of depression before and after the intervention; however, no study conducted additional long-term follow-up assessments. Regarding the changes in the level of depression, 21 studies showed significant improvement in depression, whereas seven studies reported no significant changes in depression compared to the control group [12,15,18,27,28,29,33]. The studies that failed to demonstrate a significant improvement in the level of depression were the ones that targeted only healthy adults and the ones that conducted “viewing or walking in the forest” activities only for the intervention group.

Regarding the differential pattern of findings associated with research design, while 8 out of 11 crossover trials and 8 out of 11 non-equivalent control group design studies reported significant improvement in depression scores, five out of six RCTs reported significant results. However, the differential patterns of findings could be partly attributable to the sample characteristics of the studies; four out of six RCTs were conducted with adults with health problems.

### 3.3. Quality Assessment

**Figure 2:**
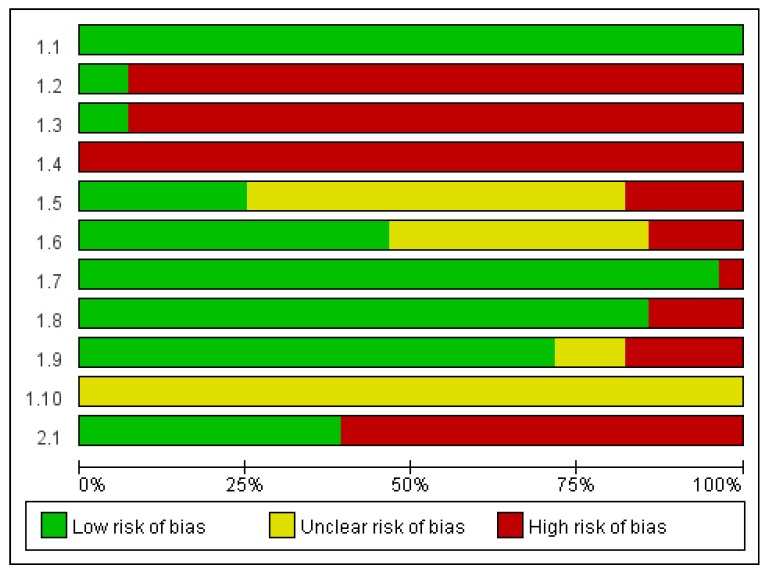
Risk of Bias Graph. Note: Authors’ judgments about each risk of bias item presented as percentages across all included studies. 1.1 The study addresses an appropriate and clearly focused question; 1.2 The assignment of participants to treatment groups is randomized; 1.3 An adequate concealment method is used; 1.4 The design keeps participants and investigators “blind” about treatment allocation; 1.5 The treatment and control groups are similar at the start of the trial; 1.6 The only difference between groups is the treatment under investigation; 1.7 All relevant outcomes are measured in a standard, valid, and reliable way; 1.8 What percentage of the individuals or clusters recruited into each treatment arm of the study; dropped out before the study was completed? (<20% = low risk of bias); 1.9 All the participants are analyzed in the groups that they were randomly allocated (often referred to as intention to treat analysis); 1.10 Where the study is carried out at more than one site, results are comparable for all sites; 2.1 How well was the study done to minimize bias?

Based on the SIGN checklist, ten out of the twenty-eight studies [16,18,19,20,21,23,25,30,34,37] met an “acceptable quality” rating and the rest eighteen studies were rated as low quality [12,13,14,15,17,22,24,26,27,28,29,31,32,33,35,36,38,39]. Six studies [16,18,19,20,21,22] used random allocations; however, no detailed description of the procedure was provided except two studies [18,20]. Five out of six RCT studies included in this review met the criteria for the “acceptable quality” [16,18,19,20,21], meeting all the items in the checklist except the criterion of blinding the treatment allocation to participants/researchers. One of the RCTs was rated “low” in terms of quality because the homogeneity between the experimental group and the control group at baseline was not ensured and the significant differences between the two groups had not been adequately addressed [22]. Three out of eleven crossover trials [23,25,34] were rated “acceptable” in terms of quality because they had low drop-out rates and the only difference between the experimental and control groups was the treatment under investigation. The main reasons for the “low quality” ratings were inadequate random allocation or method of concealment used. Among 11 categories in the SIGN checklist, studies were rated to have “high risk of bias” particularly for three categories: “the assignment of participants to treatment groups is randomized,” “an adequate concealment method is used,” and “the design keeps participants and investigators ‘blind’ about treatment allocation.” Please see Figure 2 for risk of bias graph.

## 4. Discussion

As a result of the extensive literature review, we could identify 28 studies meeting the criteria for the present review. All the studies were data-based, intervention studies with at least one comparison group. Moreover, most studies (24 out of 28 studies) were published within the last five years, confirming that forest therapy is one of the emerging therapeutic approaches and it has been gaining popularity. An analysis of the 227 regional healthcare program plans proposed in Korea between 2011 and 2014 also revealed that 35 healthcare programs were utilizing forest resources [40]. These findings demonstrated that forest therapy is a fast-growing treatment approach used in the community.

All 28 studies varied in terms of their sample characteristics and intervention types such as format, content, and study settings. Regardless of the wide variations, in general, the studies demonstrated that forest therapy is effective in improving depression, particularly for adults with health problems. However, programs that targeted only healthy adults and the ones that used “viewing or walking in the forest” activities as the only main intervention were not effective in improving depression. Per Stigsdotter et al. forest therapy is classified into three different levels of contact: “viewing nature,” “being in the presence of nearby nature,” and “active participation and involvement with nature [41].” All the activities had a certain amount of health benefits [3]; however, this review revealed that “viewing nature” or “being present near nature” may not be enough to have a significant impact on the level of depression. Therefore, future studies testing the effects of forest therapy need to include a higher level or dosage of therapeutic component of forest therapy. Another possible reason for the not-significant effect of forest therapy on improving depression in healthy adults is ceiling effect [42]. For future studies, well-thought-out intervention contents and careful selection of the outcome measures and target population are recommended. It is also important to develop structured and theory-based forest therapy programs based on the scientific evidence for the specific health benefits of forests.

Despite the increasing number of studies testing the effects of the forest therapy, these published studies are still lacking methodological rigor, mainly due to a small sample size and not having an RCT design. The majority of the studies used either non-equivalent control group design or crossover design. Crossover design, or within-subjects design, increases statistical power and enables researchers to test the effect of the intervention with relatively small samples compared to studies using between-subject design. In addition, the internal validity of crossover designs is not influenced by random assignment or between-subject variation [43,44]. On the other hand, crossover design has several limitations. For crossover design, carryover effects, the treatment effect that is carried over from one experimental period to the next experimental period, needs to be carefully examined. In addition, dropout or missing data could be the significant problem because each participant serves as both the intervention and control group; therefore, the amount of contribution made by one participant is relatively large [43,44,45,46]. However, carryover effects inherited in the crossover study design were not properly addressed in the reviewed studies. Only two crossover trials conducted by the same investigator mentioned washout periods. In addition, only 4 out of 28 studies mentioned dropout rates. Overall, issues associated with dropout or missing data were not discussed in the reviewed studies.

A second issue related to methodological rigor is the inadequacy of the control group/condition employed in the non-equivalent control group design studies. The majority of the non-equivalent control group studies used “usual care” for the control group and did not properly address factors that may threaten the findings’ validity such as the Rosenthal effect. In the future, well-designed studies with structurally equivalent control groups are needed to improve the quality.

Another shortcoming of these studies was the lack of reliable measures for assessing the level of depression. About half of these studies used self-report, paper-and-pencil-based questionnaires that only assessed the level of depression. Since the significant correlations between the physiological findings (e.g., electroencephalogram asymmetry) and the level of perceived depression has received attention [47,48], scientists have begun to use various physiological measures to assess depression in addition to self-report questionnaires. Heart rate variability (HRV) was one of the commonly used measures in the studies included in the present paper. HRV is a physiological marker that reflects the functioning of the sympathetic and parasympathetic nervous system and is also a well-established indicator of stress and depression [49,50]. A significantly reduction in HRV has been observed among patients with depression compared to the healthy adults [51,52].

Other physiological measures that have been used to assess the level of stress and psychological conditions, including depression, natural killer cell activity [53], salivary amylase activity [54,55], salivary and serum cortisol, immunoglobulin A (IgA) concentrations [56], and urinary adrenaline levels [57]. In addition, electroencephalogram-based biomarkers (i.e., rACC theta, LDAEP, iAPF, P300, frontal theta activity) were found to predict the prognosis of the course of mental illness and treatment response [58]. Therefore, future studies examining the effects of forest therapy on depression need to use well-established and reliable physiological measures in addition to self-reported questionnaires to capture the full picture of the therapeutic effects of forest therapy.

Lastly, sample characteristics of the reviewed studies deserve mention. The majority of the reviewed studies targeted healthy adult participants; only three studies tested the effects of forest therapy on adults diagnosed with major depressive disorder. Therefore, the extent that the results are applicable to clinical depression is still uncertain. More studies with clinical samples are needed to establish evidence of the therapeutic value of forest therapy. Furthermore, longitudinal studies testing the long-term effects of forest therapy on depression and the changes in depressive symptoms over a span of time are needed.

A limitation of this systematic review is language bias since we only included studies that were published in English and Korean. Studies published in other languages, such as Chinese and Japanese, were not included in this review. Despite this limitation, this study increased the understanding of the therapeutic benefits of forest therapy and identified gaps in the literature.

## 5. Conclusions

This review demonstrated that forest therapy is an emerging and effective intervention for decreasing adults’ depressive symptoms. However, the studies included in this review lacked methodological rigor. Future studies assessing the long-term effects of forest therapy on depression using rigorous study designs are needed.

## Figures and Tables

**Table 1:** General Characteristics of Included Studies (N = 28).

Variables	Categories	N (%)
Participants	Healthy adults Adults with health problems	16 (57.1) 12 (42.9)
Publication year	≤2000 2001–2011 2012–2016	1 (3.6) 3 (10.7) 24 (85.7)
Country	Republic of Korea Japan China United Kingdom	17 (60.7) 7 (25.0) 3 (10.7) 1 (3.6)
Study Design	Randomized control trial design Non-equivalent control group design Crossover trial design	6 (21.4) 11 (39.3) 11 (39.3)
Sample size	≤20 21–50 51–100 >100	12 (42.9) 6 (21.4) 10 (35.7) 0 (0)
Setting	Community Hospital University Unknown	8 (28.6) 5 (17.9) 9 (32.1) 6 (21.4)
Ethical consideration	Yes No	20 (71.4) 8 (28.6)

**Table 2:** Summary of Included Studies for Healthy Adults (**N** = 16).

Authors (Year)	Country	Research Design	Participants (N)		Intervention(s)	Control	Measurements		Outcomes
			Exp.	Cont.					
							Self-Report Measures	Physiological Measures	
Bang (2016) [18]	Republic of Korea	RCT	Office workers (n = 45)		Urban forest-walking program—Twice a week for five weeks—Urban forest walking (40 min) and Rest (10 min)	Normal daily routines	Health-Promoting Lifestyle Profile (HPLP) II Beck Depression Inventory (BDI) General Health Questionnaire/Quality of life (GHQ/QL-12)	Blood pressure (BP)	Physical activity level * Health-Promoting behavior * Depression Quality of life *
			n = 18	n = 27					
									BP
Han (2016) [24]	Republic of Korea	Non-equivalent control group design	Full-time employees from a public organization (61)		2-day forest therapy camp ^†^	Normal daily routines	BDI The Euro Qol Visual Analog Scale (EQ-VAS)	Heart Rate Variability (HRV) Natural Killer Cell (NK cell)	Self-rated health condition Depression * Ani EQ-VAS *
									HRV NK cell *
			n = 33	n = 28					
Horiuchi (2014) [25]	Japan	Crossover trial	Healthy adults (n = 15)		Viewing the forest while seated on a comfortable chair for 15 min—Switched sites with 30 min interval	Conducted same activity in an enclosed condition	Profile of Mood States (POMS)	BP HRV Salivary Amylase (sAMY)	Tension-anxiety * Depression * Anger-hostility Fatigue * Confusion * Vigor
			n = 15						
									BP * HR *, HF *, LF/HF * Salivary Amylase
Ji (2012) [26]	Republic of Korea	Crossover trial	Healthy male adult (n = 12)		Viewing (15 min) and walking (25 min) in the forest—Switched sites with 24 h. interval	Conducted same activities in the urban	POMS	BP HR Amylase concentration	Tension-Anxiety * Depression * Anger-hostility * Fatigue * Confusion * Vigor
			n = 12						
									BP * HR * Amylase concentration *
Kim (2012) [27]	Republic of Korea	Crossover trial	Healthy students (n = 50)		Forest healing program—Viewing (15 min) and walking (15 min) in a forest park landscape—Switched sites with 24 h. interval	Conducted same activities in the urban forest	POMS Semantic Differential (SD) method	None	Tension-anxiety Depression Anger-hostility * Fatigue Confusion * Vigor * Total moods disturbance * Emotion (Pleasant *, Natural *)
			n = 50						
Lee (2011) [28]	Japan	Crossover trial	Healthy students (n = 12)		Forest bathing—Viewing the forest (15 min)—Switched sites with 24 h. interval	Conducted same activity in the urban	POMS SD method	HRV Salivary Cortisol Pulse Rate (PR) BP	Emotion * Tension-anxiety * Depression Anger-hostility Fatigue * Confusion * Vigor * Total moods disturbance *
			n = 12						
									Parasympathetic nervous activity * Sympathetic activity * Salivary cortisol * PR *
Lee (2014) [29]	Japan	Crossover trial	Healthy students (n = 48)		Forest therapy program—Forest walking (12–15 min), self-paced walking in the forest environments—Switched sites with 24 h. interval	Conducted same activities in the urban	SD method The feeling of refreshed questionnaire POMS The Spielberger State-Trait Anxiety Inventory (STAI) questionnaire	HRV BP	Emotion * Tension-anxiety * Depression Anger-hostility * Fatigue * Confusion * Vigor * State anxiety level *
			n = 48						
									ln(LF/HF) * HR * BP
Lim (2014) [30]	Republic of Korea	Non-equivalent control group design	Senior citizens in a nursing home (n = 64)		Forest therapy program—Once a week (for 90 min) for 11 weeks—Meditation—Experiencing forest	Cont. 1: Indoor therapy (Conducted same activities program in the room) Cont. 2: Normal daily routines	Self-esteem Depression	None	Self-esteem * Depression *
			n = 22	Cont. 1 n = 21 Cont. 2 n = 21					
Mao G.X. (2012) [19]	China	RCT	Healthy students (n = 20)		Forest bathing—Twice a day for two days—Walking in the forest area (for 90 min), with a 10-min rest during the walk	Conducted same activities in the city area	POMS	BP Cytokine Enzyme-linked immunoassay	Tension-anxiety * Depression-dejection * Anger-hostility * Fatigue-inertia * Confusion-bewilderment Vigor-activity *
			n = 10	n = 10					
									BP * Cytokine: IL-6 * Enzyme-linked immunoassay: Renin *, AGT *
Shin (1996) [31]	Republic of Korea	Non-equivalent control group design	Students (BDI scores: 18–30) (n = 64)		5-day forest program—Group presentation, team exercise, hiking, and climbing the mountain	Normal daily routines	BDI	None	Depression *
			n = 32	n = 32					
Song (2011) [32]	Japan	Crossover trial	Healthy male adults (n = 18)		Walking in the urban forest (20 min)—Switched sites with 24 h. interval	Conducted same activity in the urban area	POMS STAI The Symptom Checklist (SCL-90) Type A behavior	None	Tension-anxiety Depression * Anger-hostility * Fatigue * Confusion Vigor * Total mood disturbance * State-anxiety * Hostility * Anxiety * Obsessive-compulsive * Somatization *
			n = 18						
Song (2014) [33]	Republic of Korea	Non-equivalent control groupdesign	Female nursing college students (n = 53)		Forest Healing Program—Once a week (for 3 h) for 12 weeks—Forest meditation—Natural healing play—Stress management	Normal daily routines	Stress Response The Spiritual Assessment Scale	None	Tension * Attack * Somatization Anger Depression Fatigue Frustration * Total points of stress responses * Total points of spirituality *
			n = 27	n = 26					
Song (2015) [23]	Japan	Crossover trial	Healthy students (n = 11)		Walking in the forest in the morning (for 15 min) Viewing the forest (for 15 min) in the afternoon—Switched sites with 24 h. interval	Conducted same activities in the urban area	SD method An inventory for the measurement of self-reported stress and arousal POMS STAI	HRV	Emotion (Comfortable, Relaxed *, Natural * Feeling refreshed *) Tension-anxiety * Depression * Anger–hostility Fatigue * Confusion Vigor * Anxiety *
			n = 11						
									Overall mean ln(HF) * Overall mean HR *
Takayama (2014) [34]	Japan	Crossover trial	Healthy students (n = 45)		Forest bathing—Walking in the forest in morning (15 min)—Viewing the forest in the afternoon (15 min)—Switched sites with 24 h. interval	Conducted same activities in the urban area	POMS Positive and Negative Affect Schedule (PANAS) The Restorative Outcome Scale (ROS) The Subjective Vitality Scale	None	Tension–anxiety * Depression * Anger–hostility Fatigue Confusion * Vigor * Negative affect * Positive Affect * The ROS score * The total SVS score *
			n = 45						
Yang (2011) [35]	Republic of Korea	Non-equivalent control groupdesign	Alcoholics’ families (n = 46)		6-day forest program—Forest experience—Drawing a forest—Day and Night walk	Normal daily Routines	Spiritual health Inventory (SHI) BDI The Rosenberg Self-Esteem Scale	None	Spiritual health * Depression * Self-esteem *
			n = 24	n = 22					
You (2014) [36]	Republic of Korea	Non-equivalent control group design	Healthy females (n = 20)		Sallimyok (Forest Therapy) Meditation Walking Qi-Qong program	Normal daily routines	Zung Self-Rating Depression Scale Psychological Well-Being Scale	None	Depression * Psychological well-being *
			n = 10	n = 10					

Note: Exp.: Experimental group, Cont.: Control group; * Significant finding; 2-day forest therapy camp; ^†^ consisted of walking, therapeutic activities, psychoeducation for coping with pain and stress, bodily exercises and mindfulness-based meditation in the forest and indoor music therapy.

**Table 3:** Summary of Studies for Adults with Health Problems (N = 12).

Authors (Year)	Country	Research Design	Participants (N)		Intervention	Control	Measurement		Outcome
			Exp.	Cont.			Self-Report Measures	Physiological Measures	
Barton (2012) [12]	UK	Non-equivalent control group design	Adults with a mental health problem (n = 53)		Green exercise Walking in the green spaces (45 min)	Cont. 1: Swimming Cont. 2: Social activities	The Rosenberg Self-Esteem Scale POMS	None	Self-esteem * Overall mood
			n = 24	Cont. 1 (n = 14) Cont. 2 (n = 15)					
Choi (2014) [37]	Republic of Korea	Non-equivalent control group design	Cancer patients (n = 53)		Forest-experience-integration intervention ^†^—Once a week (for 120 min) for 8 weeks	Normal daily routines	Zung Self-Rating Depression Scale Self-regulation Resilience	None	Depression * Resilience *
			n = 26	n = 27					
Chun (2016) [20]	Republic of Korea	RCT	Chronic stroke patients (n = 59)		4-day forest therapy program—Meditation, Experiencing the forest through all five senses—Walking in the forest	Stayed in a hotel and participated in meditation and walking activities	BDI Hamilton Depression rating scale (HAM-D17) STAI	None	Depression * Anxiety *
			n = 30	n = 29					
Hong (2012) [13]	Republic of Korea	Crossover trial	Psychiatric outpatients (n = 16)		3-day forests healing program ^‡^for patients with Hwa-Byung—Washout period (28 days)	Conducted regular diet and exercise program (3 times a day) in the forest	The Instrument of Oriental Medical Evaluation for Hwa-Byung BDI STAI State-Trait Anger Expression Inventory (STAXI) WHO Quality of Life Scale Abbreviated Version (WHOQOL-BREF)	Heart Rate Variability	Hwa-Byung symptoms * Depression * Anxiety * State anger * Trait anger Quality of Life *
			n = 16						
									Mean HR * Heart rate variability LF/HF
Hong (2013) [14]	Republic of Korea	Crossover trial	Psychiatric outpatients (n = 15)		3-day forests healing programs ^‡^ for cognitive improvement—Washout period (28 days)	Conducted regular diet and exercise program (3 times a day) in the forest	BDI STAI STAXI WHOQOL-BREF	None	Depression * Anxiety Anger Quality of Life *
			n = 15						
Jia (2016) [21]	China	RCT	COPD patient (n = 20)		One-day forest bathing trip—Forest walking in the morning (for 90 min) and in the afternoon (for 90 min)	One-day trip for urban walking	POMS	Cortisol Epinephrine	Tension-anxiety * Depression-dejection * Anger-hostility * Fatigue-inertia Confusion-bewilderment Vigor-activity
			n = 10	n = 10					
									Cortisol * Epinephrine *
Kim (2015) [15]	Republic of Korea	Non-equivalent control group design	Psychiatric inpatients (n = 20)		Forest experience program—5 times in 2 weeks (60 min each time)—Handkerchief dyeing—Decorating a frame using natural items	TAU	Korean Version of Profile of Mood State-Brief (K-POMS-B) BDI	Salivary cortisol	Tension-anxiety Depression Anger-hostility Fatigue Confusion Vigor Total moods disturbance
			n = 10	n = 10					
									Cortisol *
Kim (2015) [38]	Republic of Korea	Non-equivalentcontrol groupdesign	Cancer patients (n = 53)		Forest activity intervention—4 h. a day for 3 days—Experiencing feeling (1st day), meditation (2nd day), mindfulness (3rd day) and feedback	Normal daily routines	Hospital Anxiety and Depression Scale (HADS) Profile of Mood States-Brief (POMS-B) Dispositional Hope Scale	None	Tension * Anxiety * Depression * Anger * Fatigue * Confusion * Vigor Total mood disturbance * Hope
			n = 27	n = 26					
Mao (2012) [22]	China	RCT	Elderly with hypertension (n = 24)		Forest bathing—Twice a day for 7 days—Walking in the forest area for 90 min, with a 10-min rest during the walk	Conducted same activity in the city area	POMS	Serum Cortisol	Tension-anxiety Depression-dejection * Anger-hostility * Fatigue-inertia * Confusion-Bewilderment * Vigor-activity *
			n = 12	n = 12					
									Serum Cortisol *
Shin (2012)[16]	Republic of Korea	RCT	Adult alcoholics (n = 92)		9-day forest therapy camp ^§^	Normal daily routines	BDI	None	Depression *
			n = 47	n = 45					
Song (2015) [39]	Japan	Crossover trial	Middle-aged hypertensive individuals (20)		Walking in the forest area (for 17 min)—Switched sites with 24 h. interval	Conducted same activity in the urban area	SD method POMS	HRVHR	Emotion Tension–anxiety * Depression * Anger–hostility * Fatigue * Confusion * Vigor *
			n = 20						
									Ln(HF) * HR *
Woo (2012) [17]	Republic of Korea	Non-equivalent control groupdesign	Patients with major depressive disorder (n = 81)		Forest therapy —Once a week for 4 weeks (for 3 h)—Cognitive behavior therapy—Forest meditation—Relaxation training—Forest explanation	Cont. 1: conducted the same intervention in a hospital Cont. 2: forest bath Cont. 3: TAU	HRSD Montgomery-Asberg Depression Rating Scale (MADRS) BDI Short Form Health Survey Questionnaire (SF-36)	HRV	Depression * Quality of life *
			n = 28	Cont.1 N = 21 Cont. 2 N = 17 Cont. 3 N = 15					HF power * LF/HF ratio

Note. Exp.: Experimental group, Cont.: Control group; TAU: Treatment-as-usual; * Significant finding; ^†^ three-day forest-experience-integration intervention consisted of preparation phase (30 min), physical intervention (20 min), psychological intervention (20 min), physical intervention (20 min), and completion phase (30 min).; ^‡^ three-day forests healing program conducted in the healing forest area and consisted of forest healing activities and oriental medicine treatments. Forest healing activities included various activities in the forest, such as exercise, Qi-Qong program, and experiencing forest using five senses. Oriental medicine treatments included natural herbal diet, herbal footbath therapy, aroma therapy, herbal tea therapy, and oriental medicine music; ^§^ nine-day forest therapy camp consisted of three sessions and each session lasted for three days. Each session included various therapeutic activities including nature games and nature interpretation (1st session); mountain-climbing, trekking, and orienteering (2nd session); nature-meditation and counseling in forest environment (3rd session).

## Appendix A

**Table A1:** Search Terms Used to Identify Relevant Studies for the Review.

Forest Therapy and Depression
**Intervention**
Trees */ Tree/ Forests/ Forest/ Forest Areas/ Area, Forested/ Areas, Forested/ Forested Area/ Woodland/ Woodlands/ Forestlands/ Forestland/ Wood/ Woods/ Shinrinyoku/ Green exercise/ 1 OR 2 OR 3 OR 4 OR 5 OR 6 OR 7 OR 8 OR 9 OR 10 OR 11 OR 12 OR 13 OR 14 OR 15 OR 16 OR 17
Outcome
18. Affect/ 19. Affects/ 20. Mood/ 21. Moods/ 22. Depression/ 23. Depressions/ 24. Depressive Symptoms/ 25. Depressive Symptom/ 26. Symptom, Depressive/ 27. Symptoms, Depressive/ 28. Emotional Depression/ 29. Depression, Emotional/ 30. Depressions, Emotional/ 31. Emotional Depressions/ 32. Emotions/ 33. Emotion/ 34. Regret/ 35. Regrets/ 36. Feelings/ 37. Feeling/ 38. Depressive Disorder/ 39. Depressive Disorders/ 40. Disorder, Depressive/ 41. Disorders, Depressive/ 42. Neurosis, Depressive/ 43. Depressive Neuroses/ 44. Depressive Neurosis/ 45. Neuroses, Depressive/ 46. Depression, Endogenous/ 47. Depressions, Endogenous/ 48. Endogenous Depression/ 49. Endogenous Depressions/ 50. Depressive Syndrome/ 51. Depressive Syndromes/ 52. Syndrome, Depressive/ 53. Syndromes, Depressive/ 54. Depression, Neurotic/ 55. Depressions, Neurotic/ 56. Neurotic Depression/ 57. Neurotic Depressions/ 58. Melancholia/ 59. Melancholias/ 60. Unipolar Depression/ 61. Depression, Unipolar/ 62. Depressions, Unipolar/ 63. Unipolar Depressions/ 64. Sadness 65. 18 OR 19 OR 20 OR 21 OR 22 OR 23 OR 24 OR 25 OR 26 OR 27 OR 28 OR 29 OR 30 OR 31 OR 32 OR 33 OR 34 OR 35 OR 36 OR 37 OR 38 OR 39 OR 40 OR 41 OR 42 OR 43 OR 44 OR 45 OR 46 OR 47 OR 48 OR 49 OR 50 OR 51 OR 52 OR 53 OR 54 OR 55 OR 56 OR 57 OR 58 OR 59 OR 60 OR 61 OR 62 OR 63 OR 64
Combined terms
66. 17 AND 65
